# Small Angle X-ray Diffraction as a Tool for Structural Characterization of Muscle Disease

**DOI:** 10.3390/ijms23063052

**Published:** 2022-03-11

**Authors:** Weikang Ma, Thomas C. Irving

**Affiliations:** 1 The Biophysics Collaborative Access Team (BioCAT), Center for Synchrotron Radiation Research and Instrumentation (CSSRI), Illinois Institute of Technology, Chicago, IL 60616, USA; wma6@iit.edu; 2 Department of Biology, Illinois Institute of Technology, Chicago, IL 60616, USA

**Keywords:** muscle, X-ray diffraction, myopathy, cardiomyopathy

## Abstract

Small angle X-ray fiber diffraction is the method of choice for obtaining molecular level structural information from striated muscle fibers under hydrated physiological conditions. For many decades this technique had been used primarily for investigating basic biophysical questions regarding muscle contraction and regulation and its use confined to a relatively small group of expert practitioners. Over the last 20 years, however, X-ray diffraction has emerged as an important tool for investigating the structural consequences of cardiac and skeletal myopathies. In this review we show how simple and straightforward measurements, accessible to non-experts, can be used to extract biophysical parameters that can help explain and characterize the physiology and pathology of a given experimental system. We provide a comprehensive guide to the range of the kinds of measurements that can be made and illustrate how they have been used to provide insights into the structural basis of pathology in a comprehensive review of the literature. We also show how these kinds of measurements can inform current controversies and indicate some future directions.

## 1. Introduction

X-ray diffraction is the only technique that can provide global molecular-level structural information from striated muscle under hydrated, physiological conditions on the physiologically relevant milli-second time scale. Historically, the focus of many early X-ray diffraction studies was directed at demonstrating that crossbridges actually move axially in intact sarcomeres during contraction, as proposed in the now generally accepted “swinging lever arm” model [1,2,3,4,5,6,7,8]. X-ray diffraction studies have also played, and continue to play, a key role in understanding the structural basis of muscle regulation. Historically, some of the best evidence for the steric blocking mechanism of thin filament regulation [9,10,11] came from X-ray diffraction studies [12,13,14,15,16,17]. These studies all indicated azimuthal movement of tropomyosin on the thin filament upon binding of calcium to troponin to unblock myosin binding sites on actin. More recently it was shown that delayed stretch activation in insect flight muscle works by a similar steric blocking mechanism, in this case activated by myosin filament strain transmitted to thin filaments when sarcomeres are stretched [18]. A different mechano-sensing model has been proposed, primarily based on X-ray diffraction evidence, to be the primary regulatory mechanism for the thick filaments in vertebrate skeletal and cardiac muscle [19,20,21]. Relatively few X-ray diffraction studies have addressed relaxation, but recent studies are beginning to provide details of the temporal behavior of myosin heads after the cessation of stimulation and closing of actin binding sites [22,23]. 

While X-ray diffraction remains an important tool for investigating basic biophysical questions regarding muscle contraction and regulation, an emerging, and rapidly expanding, application is in using it for investigating the structural consequences of cardiac and skeletal myopathies. For decades, X-ray diffraction experiments were restricted to frog skeletal muscle, rabbit skeletal muscle and the indirect flight muscle of the giant waterbug, *Lethocerus*. The intense, well-collimated beams available from modern 3rd generation synchrotron sources, however, allow the study of smaller specimens such as the trabeculae and papillary muscles from wild type and transgenic small rodents that are model systems for various cardiomyopathies. Similarly, the widespread availability of mouse models for many skeletal muscle diseases has motivated recent X-ray diffraction studies of mouse leg muscles to study the structural mechanisms of contraction and relaxation in healthy muscle [24,25,26,27,28] and to investigate the pathological basis of skeletal myopathies using transgenic mouse models [29,30,31,32,33,34]. An exciting new focus, with only a relatively small number of studies so far, e.g., [30,35,36,37], is on X-ray diffraction of human skeletal and cardiac muscle. Human skeletal and cardiac muscle can yield detailed diffraction patterns suggesting that studies of muscle from human biopsies can be particularly valuable to study structural phenotypes of many muscle diseases, although only a few reports have appeared so far e.g., [36,37,38,39,40]. Finally, X-ray diffraction is beginning to be used to explore the mechanism of action of experimental drugs [41,42] on normal and transgenic porcine myocardium that is emerging as a much better model of human hearts than rodent systems. Over the last two decades, muscle diffraction has, therefore, evolved from a technique primarily used to study basic questions of muscle biophysics and physiology into an important tool in the study of the molecular mechanisms of inherited and acquired muscle diseases from nemaline myopathy to heart failure.

While X-ray diffraction can provide unique insights into important experimental systems, the interpretation of X-ray diffraction patterns is not straightforward. There are two primary approaches: (1) detailed structural modeling incorporating both X-ray diffraction and the physiological data with the goal of providing a detailed picture of the sequence of molecular events in a particular physiological protocol; (2) extraction of a limited number of biophysical parameters that can help explain and characterize the physiology and pathology of a given experimental system. The detailed modeling approach is a challenging task with many potential pitfalls [43] and best left to expert practitioners. Our contention is, however, that there are many hypotheses, particularly relevant to myopathies, that can be tested with simple and straightforward measurements that are minimally model dependent. Our goal in this review is to provide a detailed guide to the range of these simple measurements that can be made and demonstrate their utility for providing insights into the structural basis of pathology using specific examples from the literature. We also show how these measurements can inform current controversies and indicate some future directions.

### 1.1. X-ray Instrumentation and Data Analysis Software

A significant barrier to more widespread adoption of X-ray diffraction to muscle studies has been the limited availability of suitable synchrotron beamlines. At the time of writing, there are three such beamlines in the world that regularly do muscle X-ray fiber diffraction experiments, namely the BioCAT Beamline 18ID at the Advanced Photon Source (APS) in the USA, the High Flux beamline BL 40XU at Spring-8 in Japan and Beamline ID02 at the ESRF in France. Muscle studies are only a small part of the programs at 40XU and ID02, beamlines that are well-suited for muscle diffraction studies but have a primary focus on material science and soft condensed matter studies. In contrast, the BioCAT Beamline 18ID was designed and built to be optimized for small angle X-ray fiber diffraction and scattering experiments with a programmatic focus on muscle diffraction studies since it began operations in 1998. Muscle X-ray diffraction now accounts for about one third of the BioCAT user program with more than ten active groups from all over the world at any one time with a turnover rate of two-three groups per year. These experiments are facilitated by expert, on-site scientific support, a versatile suite of detectors, including a new EIGER2 X 9M pixel array detector (Dectris AG, Baden, Switzerland), and comprehensive muscle physiological apparatus. For many studies, however, multiple small-angle instruments on third and fourth generation synchrotron beamlines around the world could be used for muscle diffraction studies without major modification of the existing beamline hardware. One such example is Beamline I22 at the DIAMOND light source in the UK that was recently used for a skeletal muscle diffraction study [44]. A recent study of engineered heart tissue [45] was done on the LIXS beamline at NSLS II at Brookhaven National Laboratory (Upton, NY, USA).

Traditionally, extracting X-ray information from diffraction patterns from muscle has been a lengthy, labor-intensive task largely done by experts. BioCAT has developed a user-friendly software package, MuscleX, that enables researchers to analyze X-ray diffraction patterns without intensive training in XRD [46]. The focus in this review is on the kinds of information that can be extracted from XRD patterns rather than a tutorial on using the MuscleX software. Details on using MuscleX are provided in the user documentation [47]. In the following sections, the procedures for extracting specific kinds of information from the XRD patterns are implemented in the MuscleX software package unless otherwise stated. One of the motivations for this review is to encourage more researchers and beamlines to be involved in these kinds of studies given the availability of good analysis tools and suitable synchrotron instruments.

### 1.2. X-ray Fiber Diffraction Patterns from Vertebrate Muscle

**Figure 1:**
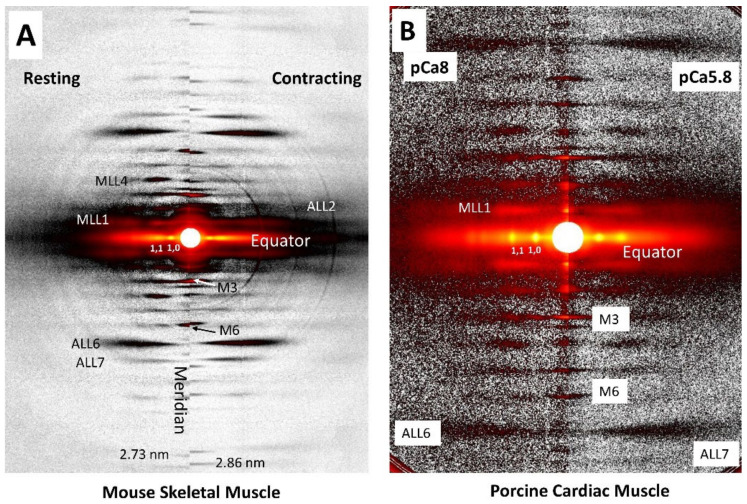
X-ray diffraction patterns. (**A**) X-ray diffraction patterns from intact mouse EDL muscle during resting (left panel) and contracting (right panel) conditions (based on reference [24]). (**B**) X-ray diffraction patterns from permeabilized porcine cardiac tissue at pCa8 (left panel) and pCa5.8 (right panel) (based on reference [49]). Panels A and B are on approximately the same intensity scale. The signal to noise is much better in the patterns shown in panel A since they are from whole intact skeletal muscle and the patterns shown in panel B are from a small cardiac fiber bundle.

Figure 1A shows a typical X-ray fiber diffraction pattern from mouse skeletal muscle and Figure 1B from porcine cardiac muscle. The general features of diffraction patterns from cardiac and skeletal muscle are similar, given that they both arise from the hexagonal array of myosin containing thick filaments and actin containing thin filaments, along with their accessory proteins, in the sarcomeres comprising the myofibrils in the muscle. Although the cellular structure of skeletal and cardiac muscle is rather different, the structure at the myofibrillar level in various muscle types is very similar so we will discuss the features of cardiac and skeletal muscle diffraction patterns together, pointing out any differences as they occur. Conventionally, these patterns are shown with the meridional portion of the pattern, representing periodicities along the long axes of the myofilaments, vertical, with the very strong equatorial reflections, arising from the hexagonal packing of the thick and thin filaments, horizontal. There are also prominent layer lines, arising from the quasi-helical packing of myosin and actin subunits in the thick and thin filaments respectively. Myosin layer lines in resting muscle will be separated by ~1/43 nm^−1^ in reciprocal (diffraction) space and the first three actin lines will be separated by ~1/36 nm^−1^. Layer lines are numbered according to their distance from the equator (MLL1, MLL2..., ALL1, ALL2…) as indicated in Figure 1. Layer lines will be located ~n/43 nm^−1^ for the nth myosin layer line and ~n/36 nm^−1^ for the first three actin layer lines. In the sections that follow, we will illustrate how each of these different diffraction features can be used to characterize and inform disease phenotypes. For further details, the reader is advised to consult Chapter 2 of Squire 1981 that provides a comprehensive overview of the physical principles governing diffraction patterns from muscle [48] and the references to the relevant literature provided in each section.

### 1.3. Equatorial Reflections

#### 1.3.1. Lattice Spacing

**Figure 2:**
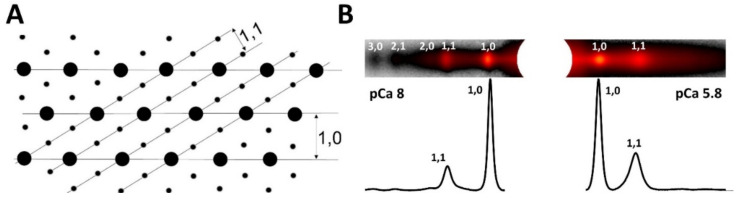
Equatorial reflections from muscle X-ray diffraction patterns (**A**) Cross-sectional view of sarcomere A band. The thick filaments (thick dots) form a 2D hexagonal lattice and the thin filaments (thin dots) interdigitate into the trigonal points between the thick filaments. (**B**) Equatorial reflections (up panel) and their one-dimensional intensity profiles (bottom panel) from permeabilized murine myocardium under resting (pCa 8) and sub-maximally activated state (pCa5.8) (based on reference [50]).

The equatorial reflections arise from the projected density of the myofilaments in the A-band of the sarcomere onto a plane as shown in Figure 2. One can draw imaginary planes through the crystallographic unit cell corresponding to various integer values of the Miller indices, h & k. The two strongest pairs of X-ray reflections in the XRD pattern of vertebrate striated muscle are the 1,0 and 1,1 reflections with intensities I_1,0_ and I_1,1_ respectively. In addition, weaker, higher-order, equatorial reflections can often be observed, as discussed in later sections.

The intensities and spacings of the 1,0 and 1,1 equatorial reflections may be determined from one-dimensional projections of the intensity along the equator (Figure 2B). The perpendicular distance between the 1,0 lattice planes, d_1,0,_ is inversely proportional to the distance of the 1,0 equatorial reflection to the beam center (S_1,0_) and can be calculated using a simplified form of Bragg’s law appropriate for small angles, d_1,0_ = λL/S_1,0_, where λ is the wavelength of the X-rays, and L is the sample to detector distance. The d_10_ lattice spacing can be converted to inter-thick filament spacing by multiplying d_10_ by 2/√3).

Lattice spacing is an important physiological parameter. Multiple theoretical studies [51,52,53,54] have shown that the amount of force that can be generated by cross-bridges is strongly influenced by lattice spacing. Implicit in these arguments is the idea that there will be a particular value of lattice spacing in a given muscle where force production is optimal [55]. Deviations from this value, due to disease causing mutations, for example, would be expected to result in less force production. While the effect of lattice spacing on force has been experimentally demonstrated many times in the literature, the relationship is not simple or direct [55]. Good examples of the effect of lattice spacing on force production are comparisons of skinned and intact muscle. When intact muscle is skinned, usually with Triton X-100, the lattice spacing swells, typically by about 15% [55]. Maximum force decreases presumably due to less favorable orientation for maximum force. When skinned, muscle is compressed by incubation with various concentrations of dextran, force will increase as it approaches the in vivo spacing and then decrease with further compression [55]. It should be noted, however, that osmotic compression with dextran appears to have consequences other than simply changing interfilament lattice spacing. For instance, osmotic compression studies of skinned rat trabeculae [56] showed abrupt, discontinuous changes in I_1,1_/I_1,0,_ over a narrow osmotic pressure range (0.7–1% dextran corresponding to 0.24–0.38 kPa) indicating a major rearrangement of myosin heads with small degrees of osmotic compression. It has been speculated but not yet shown experimentally, that osmotic compression with dextran could also alter the thick filament backbone structure which could result in altered myosin head-backbone interactions [56]. Osmotic compression also improves the ordering of the resting myosin heads as indicated by more intense myosin layer lines [57,58].

Note that fiber diameter is generally an unsatisfactory surrogate for lattice spacing measured by X-ray diffraction. Although there are situations where fiber diameter is strongly correlated to lattice spacing [59,60], in many cases inferences derived from fiber diameter can be misleading [55,61]. An example of where lattice spacing from X-ray diffraction and fiber diameter can yield contradictory conclusions is a study [62] that showed that 1% dextran was needed to compress the lattice at 1.95 μm to the same d_10_ lattice spacing as that observed at 2.25 μm whereas a previous study showed that 2.5% dextran was required to compress the fiber diameter observed at short sarcomere length to that observed at long sarcomere length [63].

#### 1.3.2. The Equatorial Intensity Ratio

Figure 2A shows the relationship of the 1,0 and 1,1 lattice planes to the filament density projected onto a plane perpendicular to the myofibril axis. The 1,0 lattice planes contain only thick filament density while the 1,1 lattice planes contain both thick and thin filament density. As a consequence, the intensity of the 1,0 equatorial reflection, I_1,0,_ arises primarily from the thick filaments, while I_1,1_ arises from both thick and thin filaments [64]. When cross-bridges bind to the thin filaments in muscle, there are radial and azimuthal movements of the cross-bridges away from the thick filament backbone so there is a loss of mass on the 1,0 planes and a gain of mass on the 1,1 planes. As a consequence, the intensities of the 1,0 reflections decrease and the 1,1 reflections increase.

It has been shown that the equatorial intensity ratio (I_1,1_/I_1,0_) is strongly correlated with force in active rodent cardiac muscle [65,66] and mammalian skeletal muscle [22,23,44]. I_1,1_/I_1,0_, under these conditions, can be used to estimate the number of cross-bridges attached to actin during contraction but since there are other factors that can affect I_1,1_/I_1,0_ (see below), one should be cautious in drawing conclusions from these estimates. I_1,1_/I_1,0_ can also be used to estimate the relative degree of association of myosin heads with thin filaments under resting conditions and has been widely used to characterize myocardium from transgenic mouse disease models, as discussed in later sections below. I_1,1_/I_1,0_ under resting conditions have also used to study the radial movement of myosin heads in the presence of various compounds [23,24,41,67], and in the presence and absence of phosphorylation of various sarcomeric proteins [68,69,70]. It should be noted that in some of the literature, e.g., [65], the equatorial intensity ratio is expressed as I_1,0_/I_1,1._ The reader needs to be aware that with this convention, I_1,0_/I_1,1_ is high in resting muscle and low when muscle contracts, the opposite behavior to that of I_1,1_/I_1,0._

While changes in I_1,1_/I_1,0_ are strongly associated with changes in radial positions of myosin heads, there are many other factors that can affect I_1,1_/I_1,0_, [71,72,73,74]. One such factor is that the flexibility of thin filaments in resting muscle may cause them to be poorly localized around their lattice positions leading to low values of I_1,1_/I_1,0_ [71,75]_._ Thus, in order to unambiguously interpret changes in I_1,1_/I_1,0_ as due to radial movements of heads and/or cross-bridge binding to actin, one needs other corroborating evidence, such as the changes in the position of layer line peak intensities described below or evidence from physiological data, e.g., stiffness. In the absence of such data, I_1,1_/I_1,0_ is still a useful operational metric to characterize a particular muscle system such as relaxed, skinned myocardium from a transgenic mouse disease model.

The conventional view of the origins of I_1,0_ and I_1,1_ given above is likely to be an over-simplification. All features in X-ray patterns arise from the entire structure at the same time so when we say that a feature is dominated by the contributions of one structural feature or another, that does not mean that other features do not contribute at all. An additional complexity is that changes in I_1,0_ and I_1,1_ have been shown to follow different time courses in a number of different muscle systems [76,77,78]. Modeling studies by the Squire group of the time courses of the 1,0 and 1,1 reflection intensities in contracting bony fish muscle suggested that the relative intensity changes of the 1,0 reflection, observed as a decrease in I_1,0_ as force increases, appeared to be a better measure of the number of attached force-producing heads than I_1__,1_/I_1,0_ [76]. Consistent with this notion, experiments of Reconditi et al. [79] on contracting frog muscle showed a linear relationship between I_1,0_ and tension. In the Eakins et al. modeling study [76], the time course of I_1,1_ appeared to best used as a measure of the initial weakly attached crossbridge population.

In order to discuss I_1,0_ and I_1,1_ independently, attention to intensity normalization is required. At this time, there are no universally agreed upon conventions for intensity normalization. It is common to use the 1,0 reflection intensity to normalize the intensities of other reflections but this approach is highly unsatisfactory since, as discussed above, I_1,0_ changes significantly more than I_1,1_ during contraction in vertebrate muscle, especially in myocardium. Other approaches that have been used in the past include dividing by the total estimated background intensity [24] and choosing a reflection that shows only small changes going from relaxed to contraction [80]. Total scattered intensity (intensity of all reflections plus the diffuse background) will be proportional to the square of the total electron density of the sample illuminated by the beam [81] and should give the most physically realistic normalization but this has not been rigorously demonstrated. Both total scattered intensity and total background intensity can be estimated by MuscleX. The popularity of I_1,1_/I_1,0_ partly arises from eliminating the need for this normalization but the reader needs to be aware of the limitations imposed by this approach.

#### 1.3.3. Characterizing Lattice Disorder Using the Equatorial Peak Widths

There is additional information in the equatorial patterns in the form of the widths of the diffraction peaks. The radial width of a given equatorial reflection, σ_hk_, where h and k are the Miller indices for the particular reflection, can be decomposed into three width parameters σ_c,_ σ_d_, and σ_s_. These parameters are interrelated by the expression σ_hk_^2^ = σ_c_^2^+ σ_dhk_ ^2^ + σ_shk_^2^ [82]. The Gaussian width of the X-ray beam at the detector is given as σ_c_ and is generally a known, fixed number. Following the notation of Yu et al. 1985 [82], σ_d_ is related to the degree of heterogeneity in inter-filament spacings in individual myofibrils as quantified by Δd_1,0_/d_1,0_^2^ where Δd_1,0_ is the standard deviation of the distribution around the mean value of d_1,0_ in the muscle sample. (Note that Yu et al., 1985 [82] omits the square on d_10_ which is dimensionally incorrect.) The distance to the first peak in the pattern is S_10_ (in nm^-1^), which is proportional to 1/d_10_. We denote the multiplicative factor f or the radial distance in reciprocal space to higher order reflections as θ_hk_ so that θ_hk_^2^ = h^2^ +k^2^ +hk. Then, we can define σ_dhk_ = σ_d_ θ_hk_ and σ_shk_ = σ_s_ θ_hk_^2^ [24]. 

The σ_s_ parameter is related to the degree of paracrystalline (liquid-like) disorder of the myofilaments in the hexagonal lattice, sometimes called “disorder of the second kind” [81]. Liquid-like disorder can result if the lattice planes containing the myofilaments slide relative to each other causing breakdown of long-range order in the hexagonal lattice. σ_s_ is defined as (π^2^/d_10_)(ΔX/d_10_)^2^, where ΔX is the standard deviation in the distribution of distances between nearest-neighbor unit cells in a given myofibril [82]. The value of σ_s_ is typically negligibly small in resting, intact wild type muscle, or relaxed, skinned, wild type muscle but can become quite large in contracting muscle [82]. It has also been observed to be larger in skinned, relaxed myocardium from transgenic mouse models [83]. Since σ_shk_ increases as the square of radial distance from the center, the effect is to smear out the higher order peaks to the point where they are indistinguishable from the background [81].

#### 1.3.4. Myofibrillar Orientation from the Angular Spread of Reflections 

In striated muscle, the equatorial reflections are perpendicular to the myofibrillar longitudinal axis; thus, the angular spread, angle σ, of the equatorial reflections across the equator (Figure 1) can be used to measure the degree of alignment of the sarcomeres in the myofibrils relative to the long axis of the preparation [40]. Angle σ can be easily estimated from equatorial patterns using the Diffraction Imaging tool in MuscleX. Angle σ has been shown to decrease with increasing sarcomere lengths in permeabilized, relaxed, wildtype mouse myocardium [40].

Myocyte disarray is a hallmark of cardiomyopathy. However, the relationship between alterations in the orientation of individual myofibrils and myofilaments to disease progression has been largely underexplored. Note that conventional histological approaches [84] measure cellular level (myocyte) orientation rather than myofibrillar/myofilament level orientation. Using angle σ from the equatorial diffraction pattern as an estimate of myofibrillar orientation avoids the artifacts introduced by conventional histological processing and provides a precise and objective metric for phenotypically characterizing myocardium [40]. This new metric may be expected to provide a generally applicable tool for establishing the relationship of myofibrillar (as opposed to the cellular level) orientation to muscle performance in future studies.

#### 1.3.5. Electron Density Maps

**Figure 3:**
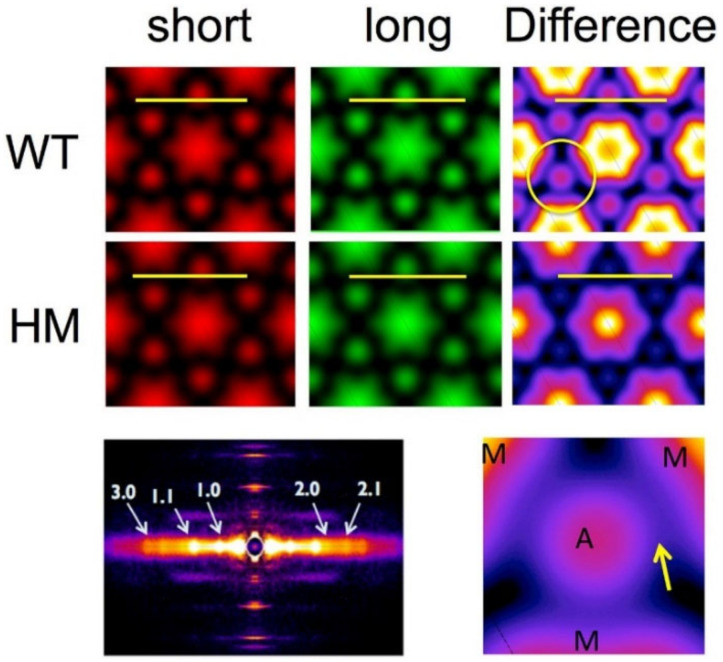
Electron density maps. Axially projected electron density maps calculated from the first five equatorial reflections. A: thin filament; M: thick filament. Calibration bar = 50 nm. Reprinted from Ref. [75]. In the difference maps, colored regions are those that have increased density at longer sarcomere length with brighter colors indicating greater increased density.

In permeabilized mouse myocardium, the 1,1 and 1,0 equatorial reflections are often the only reflections visible. In intact rat cardiac muscle and skeletal muscle additional diffraction peaks past the 1,1 are frequently visible corresponding to other, higher order diffraction planes in the crystallographic unit cell denoted by larger values of h and k. In general, the positions in the diffraction patterns of the A-band reflections obey a hexagonal lattice selection rule where S(h,k) = S_10_ * √(h^2^ + k^2^ + h * k) where S_10_ is the distance (in nm^−1^) from the center to the 1,0 reflection. In vertebrate cardiac muscle, the first 5 such equatorial reflections, namely the 1,0; 1,1; 2,0; 2,1; and 3,0, are often visible. In vertebrate skeletal muscle it is common to see the first 8 reflections, namely the 1,0; 1,1; 2,0; 2,1, 3,0, 2,2, 3,1; and 4,0. If the integrated intensities of the reflections are measured, two-dimensional electron density maps may be calculated by inverse Fourier transformation of the structure factors, quantities proportional to the square root of the integrated intensities. The structure factor is in general a complex number with an amplitude and a phase. Only the amplitudes are experimentally measurable. A simplifying assumption is usually made [71,77] that the crystallographic unit cell is centrosymmetric, at least at the low resolution of the X-ray patterns, so that the phase angles are either plus or minus π radians. A number of studies based on simple modelling and analysis of electron micrographs of transverse sections [71,77,85] have suggested that the first five reflections most likely have phases of ++−−+ but the ++−++ combination cannot be excluded [86]. It is therefore prudent to present the results from both combinations (see Ait -mou et al., 2016). At this time, there are no generally available tools to calculate electron density maps but the techniques to do so are described in [71,75,77,82,86]. The most recent application of this approach illustrating the kinds of information that can be provided from 2D electron density maps_,_ was published in Ait-Mou et al., 2016. Here electron density difference maps comparing short (2.0 μm) and long (2.4 μm) wild type rat myocardium under diastolic conditions, to short and long titin mutant myocardium expressing a long isoform of titin (LM) that results in greatly reduced passive tension [75]. Figure 3 reproduces Figure 7 of Ait-Mou et al. 2016 [75]. These difference maps clearly show the effect of titin-based passive tension on the ordering the thick and thin filaments. The thick and thin filaments tend to straighten out and become better localized around their hexagonal lattice positions when under strain when muscle is stretched in wild type muscle and this effect is greatly blunted in mutant myocardium with greatly reduced titin-based passive tension. This increases the peak density at the myofilament positions in the electron density maps providing highly complementary information the layer line and intensity ratio data. This unique ability to visualize myofilament ordering with respect to their hexagonal lattice positions suggests that this technique should be more widely used than it has been.

### 1.4. Meridional Reflections

#### 1.4.1. Overview

**Figure 4:**
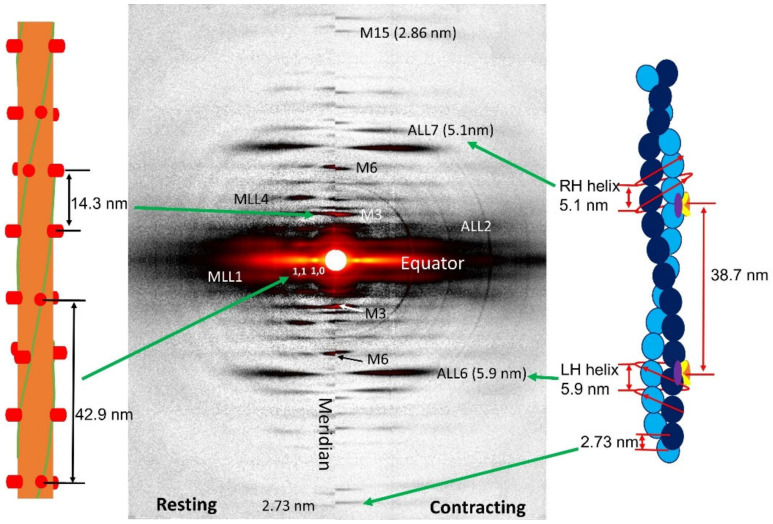
The origin of meridional and layer line reflections. Schematic diagram of the quasi-helical arrangement of myosin heads around the thick filament backbone (left panel) and the double helical arrangement of actin monomers in the thin filament (right panel). The meridional reflections arising from these features are shown in the composite X-ray diffraction pattern (based on reference [24]) in the center panel with the left half from relaxed muscle and the right from contracting mouse soleus muscle. Note that the M6 and higher order myosin meridional reflections come from structures within the thick filament backbone (not shown on the figure).

**Figure 5:**
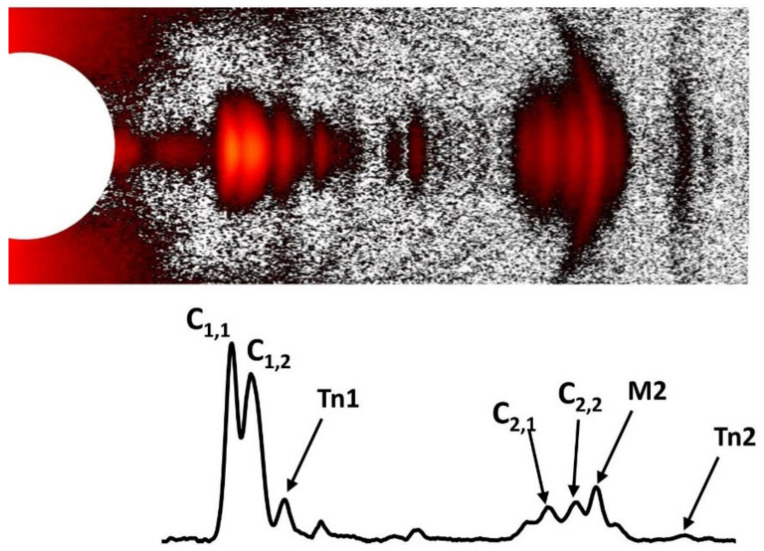
The M1 and M2 clusters in the meridional pattern (see reference [75]). The M1 and M2 clusters are located around the expected positions of the M1 and M2 “forbidden” meridional reflections. C_1,1,_ C_1.2_ and C_2,1_, C_2,2_ are doublets assumed to correspond the first order and second order MyBP-C reflections, respectively. Tn1 and Tn2 are the first and second order troponin meridional reflections, respectively. In this example, the M1 forbidden myosin reflection is not resolved and only the M2 is visible.

Meridional reflections report on axially repeating structures in the myofilaments (Figure 4). The distance of meridional reflections to the beam center in XRD diffraction are inversely related to the spacing of the axial periodicities along the myofilaments and can be measured accurately by X-ray diffraction. Myosin heads on the surface of the thick filament in relaxed muscle are arranged in a three-stranded, quasi-helical array with a 43 nm helix repeat. Myosin in resting muscle has a 14.3 nm axial periodicity along the thick filament. For a perfect three-stranded helix, meridional reflections should only be seen for every third order of the 43 nm repeat, namely the third order meridional reflection (M3 at 1/14.3 nm^−1^), the sixth order meridional reflection (M6 at 1/7.15 nm^−1^) and so on, but deviations from perfect helical order give rise to “forbidden” meridional reflections. There are clusters of reflections around the first and second order “forbidden” myosin meridional reflections at 1/43 nm^−1^ and 1/21.5 nm^−1^, the M1 and M2 cluster respectively, as well as higher order M4 and M5 reflections (Figure 5). Ait-Mou et al. showed that the M2 reflection in intact muscle increases significantly when intact rat myocardium is stretched from slack length to 2.3 µm [75]. This has also been seen in skinned porcine myocardium as a result of passive stretch [42]. This was interpreted as a titin-based strain dependent alteration in thick filament structure where the degree of helical ordering of the myosin heads in the thick filaments is reduced upon stretch and this could be a factor in myofilament length dependent activation. Analysis of the forbidden reflections have not been employed so far to characterize muscle from disease model organisms and will not be discussed further here.

There is general agreement that the intensity of the M3 reflection (I_M3_) in vertebrate muscle arises primarily from the myosin heads [43,78] while the intensity of higher order meridional reflections arises mainly from structures within the thick filament backbone [43,87,88,89]. This observation provides the basis of much of the interpretations that follow. It should be noted that the relative contributions of myosin heads and the thick filament backbone to the M3 and M6 reflections can be more complicated in invertebrate muscle [90].

#### 1.4.2. M3 Reflection

The spacing of the M3 reflection (S_M3_) in vertebrate skeletal muscle changes from ~1/14.3 nm^−1^ at rest to ~1/14.5 nm^−1^ during tetanic contraction [17,19,25,26,91,92] depending on muscle type and species. On this basis, this spacing change has been interpreted as an indicator of an OFF state to ON state transition of the thick filament in the strain dependent thick filament activation model [19], often called the “mechano-sensing” model. This is discussed further below. There is general agreement that the intensity of the M3 reflection, I_M3,_ will be high when myosin heads have a narrow distribution of angles approximately perpendicular to the thick filament backbone and low when their orientations are dispersed over many angles [5,6,93]. Since I_M3_ arises primarily from the myosin heads, the higher degree of ordering of an object, the higher the diffraction intensity. I_M3,_ therefore, has been used as one indicator of the relative ordering of myosin heads. Supporting this notion is the observed increase of I_M3_ in porcine myocardium when treated with mavacamten [42], a small molecule drug candidate that stabilizes ordered states of myosin [41]. There is some controversy [43,94] whether or not myosin heads that are out of thin filament overlap, and presumably disordered, contribute to the M3 intensity in active muscle, so it would be safest not to assume that non-overlapping heads make no contribution to I_M3_ as originally suggested by Reconditi et al. [78] until this is resolved (see also [94]).

Some of most convincing evidence for an axially moving crossbridges in intact working muscle came from X-ray diffraction studies of the fine structure in the M3 myosin meridional reflection resulting from interference between the X-rays diffracted from the arrays of cross-bridges in the two halves of each thick filament [87,95]. Analysis of the interference patterns allow determination of, with some precision, cross-bridge angles in synchronized isometric contraction after a quick release [96,97], and how these angles change with changes in load [5,79] and during isotonic contractions [98]. These conclusions depend on the details of the modeling used to interpret these findings. As a consequence, analysis of the interference pattern is beyond the scope of this review. For an alternate view concerning the interpretation of the M3 interference pattern see [43].

#### 1.4.3. M6 Reflection

The intensities of the M6 and higher order myosin meridional reflections are dominated by structures within the backbone of the thick filaments allowing them to be used as direct measurements of length changes in the thick filament backbone [99,100]. Key evidence for thick filament strain in the thick filament mechano-sensing model [19,101] is the elongation of the thick filament backbone in response to active or passive stress, as indicated by an increase in the spacing of sixth order meridional reflection (S_M6_), at low tension [19,25]. The higher order myosin- based meridional reflections (M9, M12, and M15 and so on) reflections convey the same information as the M6, but the spacing measurements from the higher order reflection will be more accurate. There is currently no general consensus in interpreting the intensity of the M6 reflection (I_M6_) other than, given that I_M6_ primarily come from thick filament backbone in vertebrate muscle, changes in I_M6_ indicate some sort of backbone structural change, whatever they turn out to be, with a relatively small contribution from the myosin heads [77,87,88,89].

#### 1.4.4. Thin Filament Meridional Reflections

The G-actin monomers in the thin filament have an axial displacement at 2.73 nm relative to each other giving rise to the 1/2.73 nm^−1^ first actin meridional reflection as well as higher order actin meridional reflections at n/2.73 nm^−1^. For many years, thick and thin filaments were assumed to be inextensible since no significant changes in periodicity were seen at the 1% level in early experiments [102,103]. In 1994, two simultaneous papers [99,100] showed that the thin filament length extended by about 0.3% from rest to the peak of isometric contraction as estimated from the spacing changes of the ~1/2.73 nm^−1^ meridional actin reflection. More recently, the Granzier group showed that the spacing of the 1/2.73 nm^-^ reflection from the thin filaments in mouse skeletal muscle is linearly related to the active tension and that the thin filament was three time stiffer when nebulin was present [29]. The linear relation of thin filament periodicity to tension suggests that this relationship can be used as a straightforward measure of thin filament extensibility in either active or contracting muscle.

One under-appreciated observation is that in frog skeletal muscle stretched beyond thick-thin filament overlap, thin filaments shorten by about 0.1% [99] when electrically stimulated. It is only when the sarcomeres develop active force do the thin filaments lengthen. These observations imply that there are structural changes in the thin filament upon binding calcium prior to developing force. Experiments reporting similar findings in mammalian skeletal or cardiac muscle have not yet appeared, but it seems that this could be fruitful avenue of enquiry for detecting structural deficits that can be related to functional defects in transgenic models or diseased muscle.

The troponin complex has an axial repeat of 38.7 nm along the thin filament, similar to, but different from the actin double helical repeat (~36 nm) [104,105,106]. The first, second, and third order troponin meridional reflections are commonly observed in the meridional XRD pattern at 1/38.7 nm^−1^, 1/19.3 nm^−1^, and 1/12.9 nm^−1^, respectively. The intensities of troponin-based reflections have been shown to change when going from resting to contracting conditions or with mutations associated with thin filament proteins [29,75,107,108,109]. These changes in the intensity of troponin-based reflections can be used to indicate a change in the structure of the troponin complex under various conditions but these low-resolution reflections cannot be used to provide molecular details of these structural changes without invoking a high-resolution structural model in computational modeling studies (see for example [110]). Tools for routinely doing such modeling are not available. In the absence of such modeling, simply knowing that the structure of the troponin complex is changing may be sufficient to make useful physiological inferences [42,75].

#### 1.4.5. Reflections from MyBP-C

Antibody labeling identified a series of meridional reflections with the first order centered around 1/44 nm^−^^1^, slightly longer than the myosin repeat of 1/43 nm^−^^1^ as coming from MyBP-C (also known as C-protein) [111]. The MyBP-C reflections appear as doublets due to interference sampling from the two sides of the sarcomere. Given that MyBP-*C* is a myosin binding protein, it would be expected to follow the 43 nm myosin repeat. Squire [112] showed that the observed longer spacing can be explained if MyBP-C was also binding actin, as is known to happen under some circumstances [113,114,115]. Given the implications for regulation, it would be highly desirable to have structural evidence for changes in the binding patterns and ordering of the MyBP-C reflections. Unfortunately, an interpretative framework for changes in C-protein spacings and intensities has not been established. The relationship between the various sub-periodicities observed on the meridian and their structural origins in the thick filaments are complex and poorly understood. Most recently, Caremani et al. [57] introduced the idea that the putative MyBP-C reflections may actually be forbidden reflections from myosin, in addition to the M1 and the M2 forbidden reflections. Because of these unresolved issues, analyses of these reflections have not yet been applied to myopathies. Therefore, a detailed discussion of the fine structure of the meridian is beyond the scope of this review.

### 1.5. Actin and Myosin Layer Lines

Myosin heads on the surface of the thick filament in relaxed vertebrate muscle are arranged in a three-stranded quasi-helix with a 43 nm helical repeat. The G-actin subunits in the thin filaments in vertebrate muscle form a double-stranded helix with approximately a 35.9 nm helix repeat (Figure 4). The arrangement of the actin subunits in the thin filaments can also be described as two families of “genetic helices” [48], one set of left-handed helices based on a helical pitch of 5.9 nm and another set of right-handed helices based on a pitch of 5.1 nm (Figure 4).

**Figure 6:**
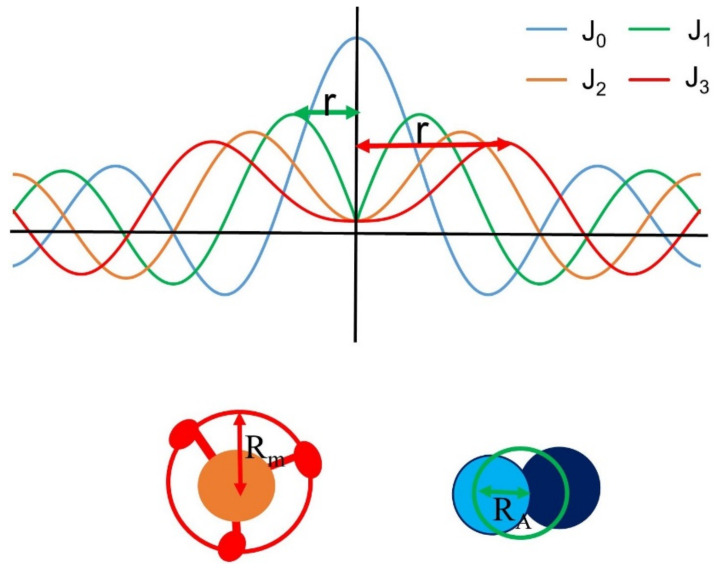
Relationship between the radius of the helical diffracting object and the distribution of intensity on a layer line. R_m_ is the radius to the center of mass of the quasi-helically arranged myosin heads and R_A_ is the radius to the center of mass of an actin subunit. J_0_–J_3_ (generally J_n_ where *n* is an integer) are Bessel functions of the first kind. The order of the Bessel function (*n*) can be determined from Equation (1) or Equation (2).

The expected diffraction pattern from a discontinuous helical structure can be described by helical diffraction theory [116]. Chapter 2 of Squire 1981 [48] provides a particularly accessible introduction to helical diffraction theory. Briefly, the diffraction pattern of a continuous helical wire will produce a series of layer lines parallel to the equator with a characteristic X-shape distribution of the intensity in the X-ray diffraction pattern. The distance between the layer lines will be inversely related to the pitch of the helices. The intensity distributions on a layer line can be described by Bessel functions of the first kind, J_n_. Bessel functions are continuous functions characterized by a series of broad peaks going radially out from the minimum (see Figure 6). The J_0_ Bessel function will have a strong peak on the meridian and a number of subsidiary peaks on either side. The order of the Bessel function is n and for a helical wire will correspond to the layer line index (*n* = 1 for the first layer line, *n* = 2 for second and so on. Note that the maxima for Bessel of functions of higher order n move progressively outwards from the meridian as shown in Figure 6. Real macromolecular structures are formed of molecular subunits, so the thick and thin filaments are examples of discontinuous helices. The axial rise between subunits will give rise to reflections on the meridian, with each meridional reflection forming the center of a cross-shaped layer line distribution (Figure 4). The superposition of these patterns gives a complex pattern of superimposed layer lines with the intensity distribution on a given layer line (Figure 6) as described by the Bessel functions present on the that layer line that are determined by the “helical selection rule” (Equation (1)).

(1) $$\frac{l}{R}=\frac{m}{p}+\frac{n}{P}$$

In Equation (1), l is the index of a layer line, an integer that increases sequentially away from the equator, m is an index for meridional reflections that increases sequentially away from the equator, n is the order of an allowed Bessel function, R is the true repeat of the helix (distance between equivalent subunits), p is the inter-subunit axial translation, and P is the pitch of helix, the distance for one turn of the helix. If one knows the number of subunits, u, in a known number of turns of helix, t, an equivalent expression is shown in Equation (2).

(2) l=um+tn

Equation (1) describes the allowed Bessel functions for a single stranded helix. Myosin containing vertebrate thick filaments are three stranded, which results in only every third layer line being visible because of destructive interference. In this case, the first visible layer line, MLL1, will have a value for l of 3, for MLL3 l will be 9 and so on.

In the relaxed state, many of the myosin heads are quasi-helically ordered along the thick filament producing strong myosin-based layer lines (MLLs) at characteristic spacings defined by the ~1/43 nm^−1^ repeat distance for the three stranded helix. This quasi-helical ordering of myosin heads is lost during contraction resulting in a reduction in the intensity of the MLL’s and an increase in the intensity of a new set of acto-myosin layer lines characteristic of contracting muscle with different axial spacings than the resting MLL’s [95]. The loss of the resting MLL’s has been proposed to indicate a transition in the myosin heads from an inactive OFF state to an ON state able to interact with actin [17,19,92,101,117]. Thus, the disappearance and reappearance of MLLs has been used to study both the activation of myosin heads during force development [19,23,44,89] and the inactivation of the myosin heads during relaxation [22,23,44,66], respectively.

#### 1.5.1. Actin Layer Lines—Spacings

Actin layer lines (ALLs) are present in the relaxed/resting XRD pattern, but under resting conditions, their intensities are generally much weaker than myosin layer lines (MLLs) except for the sixth (ALL6) and seventh (ALL7) layer lines at 1/5.9 nm^−^^1^ and 1/5.1 nm^−^^1^, respectively. During isometric contraction in frog skeletal muscle, the spacings of ALL6 and ALL7, measured most accurately from the inner part of these reflections close to the meridian, [99] show slight increases in these spacings reflecting changes in thin filament strain. However, the two periodicities do not always extend by the same proportional amount indicating that there are changes in thin filament twist when force is exerted on thin filament [99,118].

#### 1.5.2. Actin Layer Lines—Intensity Changes with Contraction

It has been known for many years that there is a general intensification of the actin-based layer lines when myosin heads bind to actin during contraction [17,119,120] and in rigor [121,122]. Because of the weakness of the actin-based reflections, most published experimental data come from quasi-static situations including resting, intact and skinned, relaxed muscle, rigor, and during the plateau of isometric contraction. There have been other attempts to model the actin layer line data from contracting muscle at full filament [123,124,125,126] with the most comprehensive being by Koubassova et al. [122]. Complications noted by that study are: (1) fine structure in the layer lines due to lattice sampling and the complex shape of the underlying filament transform; (2) the need to consider both stereo-specifically and non-stereo-specifically bound myosin heads; (3) various kinds of disorder. One of their predictions from the modeling study was that the intensity of the first actin layer line, I_ALL1,_ was an approximately linear function of the fraction of stereo-specifically bound myosin heads, with an unknown contribution from non-stereo-specifically bound myosin heads that will depend on the degree of disorder in these heads. I_ALL1_ could be a useful metric with the caveat that measurement of the first actin layer line intensity can be problematic because of its closeness to the first myosin layer line in resting muscle making it difficult to measure accurately. This situation may be expected to improve in the future with the increasing availability of larger, higher spatial resolution detectors allowing longer camera lengths. Because of the difficulty of both measuring and interpreting the intensity changes of the actin layer lines, these changes, with the exception of the “tropomyosin” reflection on the second actin layer line (see below), have not been used so far to characterize myopathies and will not be discussed further here.

#### 1.5.3. “Tropomyosin” Reflection on the Second Actin Layer Line

The textbook activation mechanism for muscle is the steric blocking model of thin filament regulation where calcium binds to troponin and initiates a cascade of events leading to force generation [11,127]. The McKillop & Geeves model [9,128] proposes that a key component of this sequence of events is movement of tropomyosin from a “Blocked” position preventing the myosin heads from binding to their binding sites on actin, to a “Closed” position allowing some heads to bind, to an “Open” position where the maximum number of heads can bind. This activation process is cooperative in that the binding of myosin heads causes changes in the tropomyosin position that allow progressively more heads to bind [129,130].

When skeletal muscle is activated, tropomyosin moves azimuthally on the surface of the thin filament, effectively changing the symmetry of the thin filament from two stranded to four stranded, resulting in an intensification of the outer part of ALL2 [48,110] that is very weak in resting muscle. For convenience, this reflection is often referred to as the “tropomyosin reflection”. This intensification is strongest in skeletal muscle in rigor when myosin binding sites on actin are maximally occupied leading to intensification of all actin-based layer lines and is weaker at low levels of activation [131,132]. The intensity of the “tropomyosin” reflection, therefore, can be used as a measure of the degree of activation of the thin filament.

There have been a number of modeling studies comparing resting and stimulated muscle at sarcomere lengths beyond thick and thin filament overlap to study activation related changes in thin filaments without the complications due to crossbridge binding [126,133,134]. Early models used spheres to represent subunits of G-actin, troponin, and tropomyosin and a more recent study by Matsuo et al. [110] used the high-resolution structures of tropomyosin and troponin core domain [135,136] docked into the Holmes fiber diffraction structure of the thin filament [137] as a starting point. All these modelling studies supported the simple picture of azimuthal movements of tropomyosin being the primary regulatory event. In addition, the modeling study of Matsuo et al. [110] required rearrangements of actin subdomains to obtain best fits to the X-ray diffraction data. More recently, Koubassova et al. [131] used cryo-EM reconstructions to construct models that provided improved fits to actin-based layer lines from rabbit skeletal muscle during relaxation and low-tension rigor. With the increasing availability of even higher-resolution cryo-EM structures for thin filaments [138,139], one can envision revisiting this kind of modeling in conjunction with time-resolved X-ray diffraction data to reveal the dynamics of these interactions, but the tools to do so will need to be developed.

No tropomyosin reflection has been reported from twitching, or even in rigor, myocardium [140].The reason for this is not clear. It is not due to the absence of nebulin in cardiac muscle since the ALL2 tropomyosin reflection is seen in insect muscle and skeletal nebulin knockout mice where nebulin is absent. Recent cryo-electron microscopy structures [138,139] suggests that tropomyosin movement in cardiac muscle may be different from the primarily azimuthal movement described above for skeletal muscle, since only the portion of tropomyosin near the troponin positions show significant axial shifts in their reconstructions, other portions appear to move much less, resulting in less change in the overall thin filament symmetry so that intensification of the ALL2 may not be expected. Further work is required to resolve this question.

#### 1.5.4. Estimating Myofilament Radius from Layer Lines

The myosin heads in the thick filaments are arranged in three quasi-helical strands around the thick filament backbone. The G-actin subunits in the thin filament are arranged approximately in a two stranded double helix (see Figure 4). Both filaments are examples of discontinuous helical arrays of subunits so the layer lines will have intensity distributions governed by the superposition of multiple Bessel functions as described by the helical selection rule defined above. Bessel functions are continuous functions characterized by a series of broad peaks going radially out from the minimum (see Figure 6). The J_0_ Bessel function will have a strong peak on the meridian and a number of subsidiary peaks on either side. Starting with the first layer line, the distance from the meridian to the first maximum on a layer line will be a function of 2π R_m_ r where R_m_ is the distance to the center of mass of the helically ordered molecular subunit and r is a radial coordinate in reciprocal space (in nm^−^^1^).

In many vertebrate skeletal muscles, the lower order layer lines are said to be “sampled”. Rather than a continuous intensity distribution on a layer line, intensity is seen only at discrete radial positions corresponding to the equatorial reflection positions in muscles with a simple lattice (e.g. bony fish muscle [141], rat soleus [142]). In most vertebrate muscles, the thick filaments do not have identical orientations but rather observe a “no three alike rule” [48,142,143]. This causes the sampling pattern on the myosin layer lines to be more complicated [48,142]. Disorder in the filament lattice reduces the effect of lattice sampling in higher order layer lines simplifying analysis. In the case of mouse skeletal muscle, the first unsampled layer line, MLL4, has been used to estimate the radius to the center of mass of the myosin heads [24] because it is well separated from actin-based layer lines. The degree of disorder in cardiac muscle is greater, so even the first myosin layer line is mostly unsampled allowing the use of MLL1 to estimate R_m_ [42,75,144]. The myosin filament is a three-stranded quasi-helix with a helical repeat of 43 nm. The effect of three helical strands is to make only every 3rd layer line visible because of destructive interference between the three strands [48]. This implies that the first visible layer line is actually the 3rd and the 4th layer line is the 12th layer line of the single strand repeat [72]. Using the helical selection rule given above, the location of the first maximum on both MLL1 and MLL4 will be for a J_3_ Bessel function with the argument 4.2 = 2 π r R_m_ allowing straightforward calculation of R_m_ using the value of r measured from the pattern providing that the layer lines are unsampled.

The ALL6 reflection is the first layer line from the left-handed genetic helix with a pitch of 5.9 nm. At low resolution, the actin structure can be described as helix with 13 monomers in 6 turns of the helix (see [48]). Using the helical selection rule, the Bessel function order n for the 6th layer line is 1. The average radius of the thin filament (R_A_) can be estimated from the radial spacing of the first maximum on the ALL6 reflection to the meridian in reciprocal space (r). The first maximum of a J_1_ Bessel function occurs when the Bessel function argument 2π r R_A_= 1.85 allowing calculation of R_A_.

## 2. Examples Where X-ray Diffraction Can Inform Questions Related to Muscle Regulation

### 2.1. The Super-Relaxed and Disordered Relaxed States of Myosin

The super-relaxed state (SRX) is defined as a state of the myosin heads that has a greatly reduced basal ATPase rate of ∼0.003 s^−1^ [49,145,146], approximately 10-fold lower than the rate observed for purified myosin in solution [145]. It has been proposed [101] that the biochemically defined SRX is equivalent to the structurally defined interacting head motif (IHM) state whereby one head of a myosin molecule binds the other thereby inactivating it [41,147,148,149]. In contrast to the ordered SRX state, there is one or more Disordered Relaxed States (DRX) where myosin heads are able to bind to actin and generate force. The strain dependent (mechano-sensing) thick filament activation model [19] proposes that force generated by a small number of constitutively active myosin heads strains the thick filament backbone leading to a large-scale structural change in the thick filament. Heads that were held in a quasi-helically ordered, sequestered state close to the thick filament backbone are released and are then able to interact with actin [101]. In the context of this model, it is tacitly assumed that there is only one structurally defined SRX and DRX state, with a one-to-one correspondence to the OFF and ON states for the myosin heads respectively. This is likely to be an oversimplification.

Assuming that only myosin heads in the SRX state are quasi-helically ordered, then all myosin layer lines would necessarily come from heads in the SRX state. If myosin heads in the SRX necessarily adopt the IHM, one would not expect to see changes in Rm, the radial position of the center of mass of the ordered myosin heads, with various experimental interventions, since the heads are assumed to be closely associated with the thick filament backbone with a fixed radius set by the IHM. In Figure 7 of reference [42], the authors show an increase in R_m_ at long SL relative to unstretched muscle indicating that there are ordered configurations for relaxed myosin heads in cardiac muscle that are not tightly packed against the thick filament backbone. In further support of this notion, these authors also showed that myosin heads at different radii, one at a smaller radius closer to thick filament backbone in the presence of blebbistatin [24] and one at a larger radius closer to actin in the presence of dATP [23], indicating that myosin heads at different radii are compatible with them being in a quasi-helically ordered state. Finally, recent FRET studies [150] showed that not all SRX heads are necessarily in the IHM state concluding that the “IHM is a sufficient but not necessary condition for SRX”.

Similarly, it appears that there is more than one state for disordered myosin heads under relaxing conditions and that disordered heads are not necessarily competent to bind to actin. Caremani et al. [26] showed that there is a state in relaxed mammalian muscle at low temperature where a population of disordered myosin heads are not able to interact with actin. More recently, studies of twitching cardiac muscle [66] indicated that only heads in the C zone of the thick filaments in resting cardiac muscle are quasi-helically ordered while those in the P and D zones are in a disordered relaxed state so that they are able to interact with actin in the presence of calcium. These are under conditions where the thick filament as a whole is in the OFF state. For these reasons, it seems unwise to refer to the disordered relaxed state (“DRX”) as one defined state. Despite these caveats, it is becoming clear that many effects of myopathic mutations are due to dysregulation of SRX/DRX transitions [151] a concept that brings new understanding to a considerable body of previous work as discussed below.

### 2.2. Myosin Binding Protein C 

Over the last two decades, there has been great interest in the structure and function of Myosin Binding Protein C (MyBP-C) in both skeletal and cardiac muscle, both from fundamental physiological and disease perspectives [152,153]. A major motivation has been the observation that heritable mutations in cardiac MyBP-C, have been linked to many cardiomyopathies, as well as dysregulation of myofilament length dependent activation (LDA) in heart failure [154,155]. MyBPC molecules are found in the C-zone on the thick filaments, corresponding to about one half the length of the thick filament. MyBPC interacts with only every third level of myosin molecules (crowns). Its C terminus interacts with the thick filament back bone and its N terminus interacts either with myosin S2 in the thick filaments or actin in the thin filaments in a phosphorylation dependent matter [69,114,115,156,157,158].

Transgenic mouse models lacking cardiac MyBP-C (cMyBP-C(−/−)) have been a useful testbed for studying the structural effects of MyBP-C. Colson et al. [83] showed that ablation of cMyBP-C in relaxed myocardium appeared to result in radial displacement of cross-bridges away from the thick filaments, as indicated by significant increases in I_1,1_/I_1,0_ accompanied by significant increases in liquid like disorder without significant changes in lattice spacing. The notion that cMyBP-C modulates cross-bridge cycling kinetics by regulating the proximity and interaction of myosin and actin was tested [69] using skinned trabeculae isolated from wild-type and cMyBP-C null mice. PKA treatment appeared to result in radial or azimuthal displacement of cross-bridges away from the thick filaments as indicated by significant increases in I_1,1_/I_1,0_ in wild type myocardium with no difference in I_1,1_/I_1,0_ between untreated and PKA-treated cMyBP-C(−/−) myocardium. There was no change in lattice spacing with pKA treatment in wildtype myocardium but treatment of cMyBP-C(−/−) myocardium significantly increased lattice spacing. This result is consistent with the idea that the myofilament lattice expands after PKA phosphorylation of cardiac troponin I, and when present, cMyBP-C, may stabilize the lattice. In a follow up study [159], X-ray diffraction of skinned trabeculae was used to elucidate the separate roles of cMyBP-C and cTnI phosphorylation in myocardial inotropy and lusitropy (the rate of myocardial relaxation). Four transgenic mouse lines were created in which the phosphorylation state of either cMyBP-C or cTnI was constitutively altered by site-specific mutagenesis. X-ray diffraction analysis indicated that cross-bridges are displaced similarly from the thick filament and toward actin (1) when both cMyBP-C and cTnI are phosphorylated, (2) when only cMyBP-C is phosphorylated, and (3) when cMyBP-C phosphorylation is mimicked by replacement with negative charge in its PKA sites. These studies support the notion that cMyBP-C acts as a tether holding myosin heads close to the thick filament backbone. If cMyBP-C is absent, or if it is phosphorylated, the tether is removed and radial motions of the myosin heads are detected as changes in I_1,1_/I_1,0_ in X-ray diffraction patterns. cMyBP-C has been proposed to stabilize the interacting heads motif (IHM) [160] implying that mutations in cMyBP-C could function by destabilizing the IHM, as indicated by reduced SRX and increased DRX in human and mouse myocardium with *MYBPC3* mutations [161,162].

Another study by Palmer et al. [68] examined the role of cMyBP-C in promoting radial and longitudinal rigidity in the sarcomeres using a different cMyBP-C knockout mode (*t*/*t*) and a constitutively unphosphorylated cMyBP-C model (AllP-(*t*/*t*)). Small-angle X-ray diffraction was used to demonstrate that ablation or dephosphorylation of cMyBP-C resulted in a more radially compressible cardiac myofilament lattice induced by rigor as compared to controls. These findings imply that the presence of WT cMyBP-C provides mechanical support for the myofilament lattice of cardiac muscle, and that the N-terminus of cMyBP-C may effectively provide a structural strut, either directly or indirectly through the myosin head, between myofilaments.

### 2.3. Myofilament Length Dependent Activation

The Frank-Starling Law of the Heart states that the force of ventricular contraction during a heartbeat is precisely regulated by the extend of end-diastolic ventricular filling, i.e., the degree of preload, established during diastole. The underlying cellular mechanism of the Frank-Starling Law involves a number of sarcomere level events collectively called myofilament length-dependent activation (LDA) [163,164,165]. Ventricular filling stretches the myofibrils thereby increasing the sarcomere length (SL). Increased SL leads to both increased sensitivity to calcium and maximum contractile force during systole, the hallmarks of LDA. LDA is perturbed in cardiomyopathic disease states, such as hypertrophic cardiomyopathy or heart failure [166,167,168]. X-ray diffraction has been an important tool in the ongoing quest to understand the structural basis of LDA [21,42,75,163,169,170,171]. At this time, the detailed mechanism underlying LDA has not been resolved [172] with seemingly contradictory findings being reported in intact rat myocardium [21,75,169] and skinned porcine myocardium [42]. For a discussion of these current controversies see reference [172]. A detailed review of the work up to this point is, therefore, beyond the scope of this review article.

## 3. X-ray Diffraction Studies of Muscle Disease

### 3.1. Introduction

The ability to perform X-ray diffraction studies on skinned trabeculae isolated from the ventricular walls of mouse myocardium has opened many rich fields of inquiry due to the ease of generating transgenic mouse models for inherited cardiomyopathies. The principal disadvantage of this preparation is that, typically, only the 1,1 and 1,0 reflections on the equator are visible, limiting inferences to those involving interfilament lattice spacing, I_1,1_/I_1,0,_ myofibrillar orientation and lattice disorder as estimated from the peak widths. Much more detail can be seen in X-ray diffraction patterns from mouse skeletal muscle preparations, rat myocardium, and skeletal muscle, as well as myocardium from porcine myocardium allowing for much more sophisticated analyses in these systems. Transgenic rat and porcine models of myopathies are relatively uncommon but are becoming increasingly available. There have been relatively few studies on human skeletal [37,39,173] and cardiac muscle [40], but the early indications are that the quality of diffraction patterns from human muscle are at least as high as from porcine myocardium, indicating that diffraction techniques can be applied to questions of direct clinical relevance. 

The equatorial intensity ratio, I_1,1_/_I,0_, has been historically interpreted as changes in the radial position of myosin so that high values of the intensity ratio are associated with heads moving away from the thick filament back bone towards the thin filaments. It now appears that at least some of these radial shifts of mass could be explained in terms of transitions between the SRX and DRX states [41,42]. As discussed above, one should not assume that there is a one-to-one correspondence of structural order to the SRX biochemically-defined state and structurally defined disorder to the DRX biochemically-defined state, so while X-ray diffraction measurements and SRX assays can be used to corroborate each other, they are not necessarily always measuring the same thing. Supporting information for head movements indicated by I_1,1_/I_1,0_ can come from estimates of R_m_, the radial distance to the center of mass of the crossbridges. More general indications of order to disorder transitions for the myosin heads can be monitored by changes in myosin layer line intensities, and the intensities of the myosin meridional reflections.

Other myopathies, especially those of skeletal muscle, appear to have their origin in dysregulated thin filaments. X-ray diffraction studies have been useful for detecting changes in the thin filament extensibility, changes in filament twist, and changes in the degree of tropomyosin movement during activation. These conclusions derive from measurements of changes in spacings and intensities of the 5.1, 5.9 nm layer lines, the 2.7 nm actin meridional, troponin reflections, and the “tropomyosin” reflection on the second actin layer line as described above.

In the sections that follow we review applications of these approaches to specific disorders of cardiac and skeletal muscle. Table 1 summarizes the studies that have been addressed

### 3.2. X-ray Studies of Cardiomyopathies

#### 3.2.1. Overview

Cardiomyopathies are genetic diseases that perturb contractile function and trigger myocardial remodeling. There are two primary distinct pathways. Hypertrophic Cardiomyopathy (HCM) shows hypertrophy of the left ventricle where systolic function is preserved and relaxation is impaired while Dilated Cardiomyopathy (DCM) is characterized by increased left ventricular volume and systolic force deficits [187]. In the sections that follow, we review studies where X-ray diffraction has played an important role in suggesting mechanisms for disease etiology. 

#### 3.2.2. HCM

HCM is a common heart muscle disease that is clinically manifested in about 1 in 500 adults [188,189]. Mutations in *MYH7* and *MYBPC3* account for 75% of cases while mutations in other sarcomere genes, namely *MYL2*, *MYL3*, *TPM1*, *TNNT2*, *TNNI3*, and *ACTC1* have been associated with ~10% of cases [188,189,190,191].

- Myosin Heavy Chain and HCM

There have been relatively few published reports so far of using X-ray diffraction to study specific myosin heavy chain based HCM mutations. One such study [41] used X-ray diffraction to characterize the effects of mavacamten, an investigational drug candidate that is currently seeking FDA approval for treating patients with symptomatic obstructive hypertrophic cardiomyopathy (HCM). The results established that mavacamten enriches the populations of heads in the ordered OFF states on the thick filament [41,42], assumed to correspond to the SRX state, in relaxed, skinned porcine myocardium as evidenced by increased intensities in the myosin-based helical layer lines and the M3 reflection, along with reduced I_1,1_/I_1,0_. The R403Q mutation in skinned transgenic porcine myocardium results in greatly reduced populations of heads in the SRX state and greatly reduced myosin layer line (MLL) intensity in X-ray diffraction patterns. Mavacamten treatment intensified the MLLs to be similar to those in resting wild type myocardium. More recently, another X-ray diffraction study demonstrated that the degree of myofibrillar orientation was compromised in R403Q myocardium [40] at a sarcomere length of 2.0 μm as evidenced by arced equatorial reflections in R403Q, as opposed to well defined spots in WT myocardium. The X-ray patterns also demonstrated greater interfilament spacing heterogeneity as evidenced by the widths of the equatorial reflections. These findings were consistent with histological studies of small rodent models of R403Q myosin-dependent HCM, which myocyte level disarray [84]. These findings suggest that measurements of myofibrillar disorientation and myofilament disarray may be useful tools for characterizing myopathies.

- Myosin Light Chain and HCM

The Szczesna-Cordary group has published a number of studies of HCM causing mutations in myosin essential and regulatory light chains where X-ray diffraction played a prominent role. One such study was on transgenic (Tg) mouse myocardium with the A57G (alanine to glycine) mutation in the cardiac ELC known to cause familial hypertrophic cardiomyopathy (FHC) [174]. Tg-A57G myocardium in rigor had a decreased interfilament spacing with no changes in I_1,1_/I_1,0_ as compared to wild type accompanied by increased stiffness in Tg-A57G. They also used the Tg-Δ43 mouse model, which expresses a truncated ELC in its myocardium, to investigate the function of the ELC N-terminal extension. Ablation of the N-terminal extension of ELC resulted in enhanced mass shift towards the thin filaments in rigor, as indicated by increased I_1,1_/I_1,0_, while not changing interfilament lattice spacing. These results support a role for the N-terminal ELC extension in prepositioning the cross-bridge for optimal force production. A similar conclusion came from earlier studies of Drosophila flight muscle with truncated RLC N-terminal extensions [192,193].

The Szczesna-Cordary group also studied mutations in the regulatory light chain (RLC), in particular the HCM related D166V (Aspartate166 → Valine) mutation which induces changes in heart morphology and function accompanied by reduced RLC phosphorylation [70]. They compared myocardium from HCM-D166V mice to that from a constitutively phosphorylated S15D-D166V mouse model. Both interfilament lattice spacing, d_10_, and I_1,1_/I_1,0_ were significantly larger in relaxed, skinned D166V myocardium as compared with WT accompanied by lower tension and a left-shifted force-pCa dependence in contracting fibers. These differences in sarcomere structure were partially or fully reversed by pseudo-phosphorylation of D166V fibers from homozygous and heterozygous S15D-D166V mice, respectively, indicating that pseudo-phosphorylated RLC in the hearts of HCM mice is sufficient to prevent the development of the HCM phenotype. In a follow up study [50], the investigators showed that both of force/pCa (Ca^2+^-sensitivity) and lI_1,1_/I_1,0_-pCa were left shifted in HCM-D166V myocardium so that over most of the submaximal activation range, cross-bridges were closer to actin-containing thin filaments facilitating activation. These results accorded with biochemical assays where the population of myosin heads in SRX were reduced indicating that SRX-to-DRX transitions are promoted in HCM-D166V mutant myocardium consistent with the phenotype of hypercontractility characteristic of HCM.

- Troponin and HCM

The Pinto group published a study [175] of how thin filament-mediated Ca^2+^ sensitization influences cross-bridge kinetics in skinned myocardium from a murine model of hypertrophic cardiomyopathy (HCM) harboring a cardiac troponin C (cTnC) Ca^2+^-sensitizing mutation, cTnC-A8V, in the regulatory N-domain. They compared the results to those from wild type myocardium in the presence and absence of bepridil, a cTnC-targeting Ca^2+^ sensitizer. Combined X-ray diffraction and mechanical experiments were employed to measure myofilament lattice spacing, I_1,1_/I_1,0_ and isometric force from skinned myocardium during steady-state Ca^2+^ activation at near in vivo lattice spacings in the presence of dextran. The results showed that lattice spacing of both control and cTnC-A8V myocardium increased with increasing Ca^2+^-activated, isometric force but not in control fibers treated with bepridil. Significantly larger lattice spacing was observed for both cTnC-A8V and control fibers treated with bepridil compared to Ctrl at submaximal activation due at least in part to increased Ca^2+^ sensitivity. On the basis of these findings, the authors proposed that the transient expansion of the myofilament lattice during Ca^2+^ activation may be one of the factors that increase the rate of cross-bridge cycling in cardiac muscle.

The Yagi group recently published a study [109] of a hypertrophic cardiomyopathy related mutant Troponin T where troponin complexes in rat left ventricular skinned myocardium were exchanged with those containing the HCM-related mutants E244D- and K247R in troponin-T. Activation of fibers decreased the intensity of the troponin meridional reflection at 1/38.5 nm^−1^ after exchanges with either wild-type or mutant troponin-T, indicating that crossbridge formation affected the conformation of troponin-T. Equatorial X-ray intensities and tension measurement of myocardium after troponin exchanges showed that E244D-TnT, show enhanced recruitment of actomyosin interaction, as evidenced by increased tension and I_1,1_/I_1,0_, while K247R-TnT did not. The authors interpreted this to imply that K247R-TnT induced abnormal contractility through disrupted linkage between the structural change of troponin and actomyosin recruitment.

- Tropomyosin and HCM

Tropomyosin (TM) is encoded by three striated muscle isoforms: α-TM, β-TM, and TPM 3. The TPM 3 isoform is mainly found in slow-twitch muscle in mammals. To study the TPM 3 isoform, the Wieczorek group generated six transgenic mouse lines that produce varying levels of TPM3 protein [194]. Analysis of skinned fiber bundles showed decreased Ca^2+^ sensitivity and a decreased LDA with increasing TPM3 with no detectable change in interfilament spacing as determined by using X-ray diffraction. This observation was seen as additional confirmation that interfilament lattice spacing could not be a unifying hypothesis to explain length dependent activation [163], (see discussion above under Myofilament Length Dependent Activation).

#### 3.2.3. DCM

Dilated Cardiomyopathy (DCM) is a common disorder with an estimated prevalence of 5–7 cases per 100,000 population [195]. Point mutations contribute to 25–50% of all DCM cases [196], and among these, mutations in the titin gene (*TTN)* are the most common with mutations in other sarcomere genes, including *MYH7, MYBPC3, TNNT2*, and *TPM1* being less common and affect different residues than mutations that cause HCM. DCM is characterized by reduced contractility, i.e., systolic dysfunction. In typical DCM, ventricular volume is increased to maintain cardiac output through the Frank–Starling mechanism, resulting in the thin-walled appearance in the left ventricle [187].

- Myosin Mutations and DCM

As an unusual approach to studying mutations in myosin, the Bernstein group [176] expressed the S532P DCM mutation in *Drosophila* as a model for human DCM. As part of their characterization of the mutant flies, they compared the X-ray diffraction patterns obtained from mutant and control indirect flight muscle (IFM) during active contraction. Lattice spacing of active S532P fibers was reduced by about 4.1 nm compared with transgenic wild-type fibers. The ratio of 1,0 and 2,0 intensity (*I*_2,0_/*I*_1,0_) was used as an estimate of shifts of molecular mass from the region of the thick filament to that of the thin filament, in analogy to I_1,1_/I_1,0_ in vertebrate muscle [64]. *I*_2,0_/*I*_1,0_ of S532P was significantly decreased relative to that of transgenic wild-type flies, suggesting that S532P myosin heads were less associated with the thin filament and fewer cross-bridges are formed during active contraction compared with controls in agreement with the muscle mechanics results, suggesting that the mutation decreases the number of bound cross-bridges which could partially explain the observed force deficits.

- Myosin Light Chains and DCM

The Szczesna-Cordary group published a study [177] where they investigated the biomechanical and structural causes of DCM using a D94A (aspartic acid-to-alanine) mutation in the cardiac myosin regulatory light chain mouse model. Small-angle X-ray diffraction patterns in D94A papillary muscle fibers at submaximal Ca^2+^ concentrations showed reduced *I*_1,1_*/I*_1,0_ compared to controls, supporting the hypocontractile state of the D94A myosin motors including a reduced calcium sensitivity of force. In a follow up study [50], X-ray diffraction patterns were collected as a function of calcium activated force to generate full I_1,1_/I_1,0_-pCa curves, but the structure/function-pCa data were similar to WT indicating that the D94A mutant does not result in a shift of the equilibrium of relaxed heads from the OFF to ON states, consistent with the observed increased SRX population relative to wild type at rest.

- Myosin Binding protein C and DCM

The Sadayappan group recently published a study [178] of a transgenic DCM mouse model expressing cMyBP-C, but lacking its C0-C1f region (cMyBP-C^∆C0-C1f^) in order to investigate the importance of the N′-region in cMyBP-C. I_1,1_/I_1,0_ in skinned papillary muscle from transgenic hearts was significantly lower than in non-transgenic controls while interfilament lattice spacing was significantly higher. Parallel biochemical assays indicated no significant difference in the proportion of myosin heads in the SRX state between these groups. These results suggest that loss of the C0-C1f region leads to a net transfer of myosin head mass closer to the thick filament backbone along with an increased interfilament spacing in resting myocardium, interestingly, without significant enrichment of the population in the SRX state.

#### 3.2.4. Muscular Dystrophy Cardiomyopathy

Duchenne Muscular Dystrophy (DMD) is associated with progressive depressed left ventricular (LV) function, but the structural bases of these deficits are not well understood. The Cazorla group did an X-ray diffraction study [179] of myocardium from a Golden Retriever Muscular Dystrophy (GRMD) dog model of DMD recapitulating the human form of DMD. They observed a negative correlation between SL and lattice spacing in both sub-epicardium (EPI) and sub-endocardium (ENDO) LV layers in control dog hearts. They found that in the ENDO of GRMD hearts, however, the lattice spacing was larger at short SL (1.9 μm) leading to a steeper curve. The kinetics of tension redevelopment (ktr) was observed to be faster in ENDO GRMD myofilaments at short SL. This unexpected finding requires further study

#### 3.2.5. Ischemia

In this study [180], the Pearson group introduced an innovative way to collect X-ray diffraction data under conditions very close to physiological, namely a rat open chest preparation where the left ventricular free wall of spontaneously beating rat hearts were examined using the high-flux, low-emittance x-ray beams at the BL40XU beamline at SPring-8, Japan. This arrangement allowed them to detect real-time changes in rat left ventricle contractility, mass shifts of myosin heads, and myosin lattice spacing, as a function of position in the heart. Note that all papers from the Pearson group reviewed here reported the intensity ratio as I_1,0/_I_1,1_ and we have converted these to I_1,1_/I_1,0_ for consistency with the other studies reviewed here. They found that mass transfer, as indicated by increases in I_1,1_/I_1,0_, during systole preceded significant increases in lattice spacing in nonischemic pericardium. Left coronary occlusion, to simulate ischemia, eliminated these increases in lattice spacing and severely reduced mass transfer, as indicated by reduced increases in I_1,1_/I_1,0_, in the ischemic region.

#### 3.2.6. Diabetic Cardiomyopathy

Diabetes mellitus significantly increases the risk of cardiovascular disease and heart failure in humans. In additions to hypertension and coronary artery disease, diabetes can lead to diabetic cardiomyopathy. Using their innovative open-chest rat preparation, the Pearson group performed a series of X-ray diffraction studies of diabetic cardiomyopathy that are reviewed in Waddingham et al. [181]. Results from early diabetic model rats [182] reported that myosin heads were displaced towards the thick filaments and away from the thin filaments throughout the cardiac cycle with I_1,1_/I_1,0_ being minimal at end diastole. This effect was largest in the deeper subendocardial layer. The expected increase in I_1,1_/I_1,0_ induced by dobutamine (β-adrenergic stimulation) was significantly reduced in diabetic animals. Closer association of myosin heads with the thick filaments was also found at end diastole in the subendocardial layer of the LV wall in their study of young prediabetic Goto-Kakizaki (GK) rat model of type II diabetes [181]. In hindsight, these results would be consistent with dysregulated SRX/DRX transitions in early diabetic cardiomyopathy.

In a later paper, the Pearson group examined the question whether diastolic dysfunction precedes coronary endothelial dysfunction and nitric oxide depletion in prediabetes in the GK prediabetic rat model [183]. In diastole, I_1,1_/I_1,0_ was reduced across the group in the prediabetic rats relative to the control rats consistently in the subendocardial layer but less so in the epicardial and subepicardial layers. As expected, I_1,1_/I_1,0_ was greater than that of relaxed myocardium (in KCl-arrested hearts) in the diastolic phase in the beating hearts of control rats, while in the prediabetic rat, it was often less than that of the relaxed state implying that heads were closer to the thick filament backbone. In systole, I_1,1_/I_1,0_ increased to a maximum value higher in the prediabetic rat hearts in both subepicardial and subendocardial layers relative to controls. Dobutamine increased myosin mass transfer towards the thin filaments, as indicated by increased I_1,1_/I_1,0_, in both control and prediabetic model rates. Together, the results showed that prediabetes induced myosin head displacement towards the thick filament backbones (reduced I_1,1_/I_1,0_) in diastole during baseline steady-state contractions in the prediabetic rats, but this was reversed with β-adrenergic stimulation so that the intensity ratios were similar in both strains. Cardiac relaxation and cross-bridge dynamics, therefore, are impaired by decreased mobilization of myosin heads in prediabetic rats.

### 3.3. Skeletal Muscle Diseases

Mutations in genes encoding myosin heavy chain can result in myosinopathies that are associated with skeletal muscle weakness or paralysis [197,198]. Most human skeletal muscle myopathies, however, are due to mutations in genes that express thin filament proteins, namely skeletal α-actin, β-tropomyosin, γ-tropomyosin, fast skeletal muscle troponin I, slow skeletal muscle troponin T, fast skeletal muscle troponin T, and nebulin. These mutations have been linked to specific myopathies including nemaline myopathy, distal arthrogryposis, cap disease, actin myopathy, congenital fiber type disproportion, rod-core myopathy, intranuclear rod myopathy, and distal myopathy, usually resulting in muscle weakness that may have severe impacts on quality of life [199].

- Myosin-based Skeletal Myopathies

Despite their prevalence, few X-ray diffraction studies have addressed myosin based skeletal myopathies. One notable paper was by the Ochala group [33] who studied the MYH4L342Q mutant in a transgenic mouse model. This mutant is in the catalytic domain of the type IIb skeletal muscle myosin protein encoded by MYH4. Their results demonstrated higher stiffness as well as altered meridional (I_M3_) and equatorial reflections (increased I_1,1_/I_1,0_) in Myh4(arl)/+ mice when compared with age-matched WT animals at maximal Ca ^2+^ activation. These results showed that the transition from weak to strong myosin cross-bridge binding is enhanced in adult MYH4 (L342Q) heterozygous mice resulting in greater maximal force.

- Titin-based Skeletal Myopathies

The Ottenheijm group used X-ray diffraction as part of a study [184] of Ehlers-Danlos Syndrome caused by a loss of tenascin-X (TNX) in the extracellular matrix. In muscle fibers from patient biopsies, *d_1,0_* decreases with increasing sarcomere length, and that this relation is shifted downward in muscle fibers from patients with TNX deficiency compared with control subjects. They attributed the reduced lattice spacing to a stiffening of titin in response to the disease condition that compensates for the muscle weakness in these patients by augmenting submaximal active tension generation via a smaller lattice spacing. The same group also studied facioscapulohumeral muscular dystrophy (FSHD) in muscle biopsies from FSHD patients [38]. FSHD fibers produced only 70% of active force compared to healthy controls, a reduction which was exclusive to type II muscle fibers. Passive force was increased 5- to 12-fold in both fiber types, with increased calcium sensitivity of force generation and decreased myofilament lattice spacing, which they interpreted as indicating compensation to FSHD by titin.

- Nemaline Myopathies

Nemaline myopathy (NM) is one of the most common congenital non-dystrophic myopathies and is characterized by severe hypotonia, muscle weakness, feeding difficulties, respiratory failure [200], and the presence of nemaline bodies (rods) [201]. Many forms of nemaline myopathy arise from mutations in nebulin, a giant protein that winds around the actin filaments in the sarcomeres of skeletal muscle. Absence or reductions of nebulin expression is associated with severe nebulin-based NM [202]. To examine the effects of the absence of nebulin on sarcomere structure, the Granzier group [29] studied resting and contracting skeletal muscle from an inducible nebulin-knockout mouse model. By examining changes in the spacing of the actin subunit repeat (~2.7 nm) during contraction, thin filaments were found to be threefold less stiff in nebulin-knockout muscle while thick filament stiffness was unaffected. Measurements of the A6 and A7 layer lines revealed a significantly shorter left-handed (5.9 nm) thin filament helical pitch, with no change in the shorter (5.1 nm) right-handed helix in passive and contracting Neb cKO muscles, indicating a significant increase in actin filament twist with respect to wild type. The study also showed that the intensification of the “tropomyosin” reflection on ALL2 with contraction was less in the Neb cKO muscle than in WT. The intensity of the TN3 reflection was significantly reduced in resting Neb cKO muscle, increasing with contraction in WT, but decreasing in Neb cKO indicating a change in the troponin structure with respect to wild type under both resting and contracting conditions. I_1,1_/I_1,0_ was significantly lower in both passive and contracting Neb cKO muscle as compared to WT. The intensity of MLL4 was reduced during tetanic contraction due to the loss of order when myosin heads interact with the thin filament, but the reduction was much less in Neb cKO, indicating a significantly higher proportion of the ordered myosin heads remaining around the thick filament backbone during contraction in the absence of nebulin. All these results support the notion that nebulin is required to recruit sufficient numbers of crossbridges to generate physiological levels of force in skeletal muscle.

Typical, less severe, nemaline myopathy (NM), is caused by mutations in the nebulin gene (*NEB*) rather than the absence of nebulin. To approach this problem, the Ochala group [37] studied biopsies from a human patient who had compound heterozygous mutations, one leading to skipping exon 3 and the other exon 22 of the nebulin gene. This technically impressive study used arrays of 20 single skinned muscle fibers to yield excellent quality X-ray diffraction patterns. At full thin-thick filament overlap sarcomere lengths, ALL6, ALLL5 and the tropomyosin reflection intensified, as expected, in controls on addition of calcium while the intensification of the troponin reflection was blunted in patient fibers. When fibers were stretched beyond thin-thick filament overlap, the tropomyosin reflection increased during contraction, similarly in the controls as in the patient, indicating that the deficit at full overlap was probably due to less crossbridge binding in the patient. Equatorial intensity ratio measurements indicating less transfer of myosin heads in the patient with activation. More recently, Lindquist et al. 2020 [31] studies of a Compound-Het mouse model created to resemble typical NM with a missense mutation p.Ser6366Ile and a deletion of *NEB* exon 55. In this case, the Compound-Het mice express normal amounts of nebulin. The study examined EDL muscle in its passive state before activation and during the plateau of a tetanic contraction in Compound-Het and WT mice. Measurements of the 2.7 nm reflection spacing during the tetanic force plateau were used to show that the thin filament stiffness was identical in WT and Compound-Het muscles, unlike with the nebulin KO model. However, thin filaments in the Compound-Het model muscle showed, similar to those in the nebulin KO, a significant reduction in the spacing of the left-handed (5.9 nm) thin filament helical pitch, with no change in the shorter (5.1 nm) right-handed helix, indicating a greater degree of twist of the thin filament helix than in WT muscle. This study was the first instance of using R_A,_ the distance from the filament axis to the center of mass of actin subunits. to show that when the thin filaments became shorter in Compound-Het EDL muscle, they also expanded radially (increased R_A_). Furthermore, similar to nebulin KO muscle, the intensity of the tropomyosin reflection on ALL2, and the intensity of the third meridional troponin reflection, I_TN3_, are both reduced upon activation in the Compound-Het EDL muscle. 

There are nemaline myopathies due to mutations in proteins other than nebulin. ACTA1 encodes alpha-actin 1, the main constituent of the sarcomeric thin filament and are a frequent cause of nemaline myopathies including NEM3 [203]. In a recent paper in Ann. Neurol [36] the authors used small-angle X-ray diffraction to demonstrate that there were no differences in lattice spacing or in resting I_1,1_/I_1,0_ in NEM3 patient biopsies as compared to control samples. The expected increase in I_1,1_/I_1,0_ during fiber activation, however, was markedly lower in NEM3 patients compared to the control subjects. These findings strengthened the notion that fiber activation results in less acto-myosin cross-bridge binding in fibers from NEM3 patients as compared to fibers from control subjects. A different X-ray diffraction study by the Ochala group of a mouse model for D286G mutant skeletal muscle α-actin protein came to similar conclusions [185]. At full thick-thin filament overlap, intensification of ALL6, ALL7, the tropomyosin reflection and the M3 meridional reflection were blunted in the mutant myocardium as compared to controls but were indistinguishable in overstretched muscle indicating that the replacement of just one amino acid in the skeletal muscle α-actin protein was sufficient to impair actin conformational changes and tropomyosin movement during activation that would allow strong binding of myosin molecules leading to impaired contractility. 

One form of nemaline myopathy (NEM6) that is characterized by impaired relaxation kinetics is caused by mutations in the actin-binding protein KBTBD13 [30]. X-ray diffraction patterns from human NEM6 and control fibers showed no change in lattice spacing, I_1,1_/I_1,0,_ M6, M3 spacings but the ALL6 spacing is reduced in NEM6 indicating thin filament twist relative to control fibers. In follow-up studies on a Kbtbd13-KI mouse model, they were able to use measurements of the first meridional reflection at 2.7 nm to show that thin-filament stiffness is higher in muscle of Kbtbd13R408C-KI mice than in muscle of WT mice. These results suggest that stiffer thin filaments in muscle of Kbtbd13R408C mice could be the cause of the slower relaxation kinetics in NEM6.

- Tropomyosin Based Myopathies

Human point mutations in β- and γ-tropomyosin induce contractile deregulation, skeletal muscle weakness, and congenital myopathies. To investigate disease mechanisms, the Ochala group performed X-ray diffraction experiments [186] on skinned human muscle fibers expressing the R133W β-tropomyosin mutation that has been associated with a loss in cell force production, muscle weakness, and distal arthrogryposis. With this mutation, intensity changes at full filament overlap in the presence of calcium on the ALL6, ALL7 and the tropomyosin reflection on the second layer line were blunted in the mutant with a reduction in the tropomosin reflection also observed in muscle stretched beyond filament overlap. Changes in I_1__,1_/I_1,0_ (reported as I_1,0_/I_1,1_) with calcium were similarly blunted at full overlap. Collectively, these results show that during activation, this mutation partially hinders both calcium- and myosin-induced tropomyosin movement in the thin filament and, consequently, decreases the number of strong binding cross-bridges and maximum force.

## 4. Conclusions and Future Directions

X-ray diffraction is a powerful technique that can provide structural information, often unobtainable any other way, concerning the arrangements of sarcomeric proteins under near physiological conditions. However, one must keep in mind that it is a low-resolution technique compared to other structural techniques such as X-ray crystallography, NMR, and Cryo-EM. Muscle X-ray diffraction can, therefore, only detect relatively large-scale structural changes and it is unwise to extrapolate conclusions from such studies to the sub-molecular level. It must also be emphasized that X-ray diffraction reports the average of all structures illuminated by the X-ray beam at the time of exposure so that in any given situation there are several different coexisting conformations contributing to the observed diffraction patterns. This suggests a role for multi-scale, spatially explicit, modeling approaches where specific combinations of molecular conformations can be used to predict observed structural and functional outcomes that can only reflect aggregate behavior. We have made a start on this process by developing extensions to the spatially explicit simulation platform MUSICO [204] to predict specific features of muscle fiber diffraction patterns, starting with thin filament meridional reflections [51,205,206]. In our opinion, these kinds of modeling studies are best used as a hypothesis generating tool that can reveal possible specific scenarios that may be tested by more incisive experiments. It is seldom possible with any modeling approach to prove that one has obtained a unique explanation for a given set of experimental observations [77]. As a consequence, the more disparate pieces of information that a given model can predict, the more confidence we will have in the model as it adapts iteratively to new knowledge as it comes along. 

What we have shown here, however, is that X-ray diffraction, in the absence of detailed modeling, can be used to establish structural correlates to functional observations that may help explain physiology and to phenotypically characterize mutations related to myopathies. Emerging applications of these techniques are represented in ongoing studies of heart failure in human myocardium [40] and skeletal muscle myopathies such as Duchenne Muscular Dystrophy [179] among other studies, as yet unpublished. In combination with biochemical studies, including SRX/DRX transitions, and physiological assays, information derived from X-ray diffraction studies can provide critical insights leading to a mechanistic understanding of the etiology of these diseases. 

## Figures and Tables

**Table 1:** Studies of Myopathies Using X-ray Diffraction.

Muscle Type	Disease	References
Cardiac	Heavy Chain HCM	[40,41]
Cardiac	Essential Light Chain HCM	[174]
Cardiac	Regulatory Light Chain HCM	[50,70]
Cardiac	Troponin HCM	[109,175]
Cardiac	Tropomyosin HCM	
Cardiac ^1^	Heavy Chain DCM	[176]
Cardiac	Regulatory Light Chain DCM	[50,177]
Cardiac	cMyBP-C DCM	[178]
Cardiac	Muscular Dystrophy Cardiomyopathy	[179]
Cardiac	Ischemia	[180]
Cardiac	Diabetic Cardiomyopathy	[181,182,183]
Skeletal	Myosin-based myopathies	[33]
Skeletal	Titin-based myopathies	[38,184]
Skeletal	Nemaline myopathies	[29,30,31,36,37,185]
Skeletal	Tropomyosin-based myopathies	[186]

^1^ In an insect flight muscle model.

## Data Availability

Not applicable.
